# Vanin-1 deficiency enhances host tolerance to influenza infection by modulating cellular redox status

**DOI:** 10.1093/pnasnexus/pgag222

**Published:** 2026-06-22

**Authors:** Jin Soo Joo, Lesley Pasman, Shuang Yu, Robert Homer, Philippe Naquet, Ruslan Medzhitov, Jun Young Hong

**Affiliations:** Department of Systems Biology, Yonsei University, Seoul 03722, South Korea; Department of Immunobiology, Yale University School of Medicine, New Haven, CT 06510, USA; Department of Immunobiology, Yale University School of Medicine, New Haven, CT 06510, USA; Department of Pathology, Yale University School of Medicine, New Haven, CT 06510, USA; Centre D'Immunologie de Marseille-Luminy, INSERM, CNRS, Aix-Marseille Université, 13288 Marseille, France; Department of Immunobiology, Yale University School of Medicine, New Haven, CT 06510, USA; Howard Hughes Medical Institute, Chevy Chase, MD 20815, USA; Department of Systems Biology, Yonsei University, Seoul 03722, South Korea; Department of Immunobiology, Yale University School of Medicine, New Haven, CT 06510, USA

## Abstract

Host survival during infection depends on the balance between pathogen resistance and tissue tolerance mechanisms. Although resistance pathways are well characterized, the molecular determinants of host tolerance remain poorly understood. Here, we demonstrate that vanin-1, an ectoenzyme known for its role in vitamin B5 metabolism, unexpectedly regulates host tolerance during influenza infection through modulation of cellular redox status. Mice lacking vanin-1 showed enhanced survival following lethal influenza infection without alterations in viral burden, immune responses, or tissue pathology. While vanin-1 deficiency impairs vitamin B5 metabolism, neither vitamin B5 supplementation nor deficiency affected survival during infection, indicating a vitamin B5–independent mechanism. RNA sequencing analysis revealed enhanced expression of antioxidant pathway genes in vanin-1–deficient mice, with Nrf2 serving as a key upstream regulator. Mechanistically, vanin-1–deficient mice maintained higher glutathione levels during infection and showed reduced lipid peroxidation, suggesting protection against oxidative stress-induced cell death. Importantly, pharmacological inhibition of glutathione synthesis abolished the survival advantage in vanin-1–deficient mice, while glutathione supplementation protected wild-type mice from lethal infection. Our findings reveal an unexpected role for vanin-1 in regulating host tolerance through antioxidant pathways and identify a potential therapeutic target for enhancing survival during severe viral infections.

Significance statement Host survival during infection depends on both pathogen control and tissue tolerance mechanisms. This study identifies vanin-1, a metabolic enzyme, as a regulator of host tolerance during influenza infection through control of cellular redox status. The findings establish vanin-1 inhibition as a therapeutic strategy to enhance survival in severe viral infections.

## Introduction

The relationship between diet and immune function has long been a focus of research, highlighting the critical role of nutrition in maintaining immune homeostasis and resistance against infections. Proper nutrient intake enables immune cells to develop, activate, and orchestrate effective responses against pathogens, while nutritional deficiencies can significantly impair these protective functions. Beyond serving as a source of energy, various dietary metabolites also act directly as signaling molecules, playing a role in modulating immune responses (1). Additionally, these nutrients can affect the host's ability to combat infections by maintaining tissue homeostasis or regulating stress tolerance (2).

Essential vitamins serve as critical modulators of immune function through diverse mechanisms. Fat-soluble vitamins A and D enhance immunity by promoting tissue-specific homing of immune cells and supporting proper T-cell differentiation (3, 4), while vitamins C and E strengthen immune defense by maintaining epithelial barriers and enhancing neutrophil function (5, 6). These vitamins also possess important antioxidant properties that help regulate inflammatory responses and protect against oxidative stress during infection. Within the B-complex family, vitamins B6 and B12 are well-established regulators of lymphocyte proliferation, cytokine production, and immune signaling, supporting white blood cell production, enhancing T and NK cell activity, reducing inflammation, and promoting overall immune resilience (7–9). However, the role of vitamin B5 (pantothenic acid) in immune regulation remains less understood. As a precursor for coenzyme A (CoA) synthesis, vitamin B5 is essential for cellular energy production and lipid metabolism. These metabolic processes indirectly influence immune function by maintaining the energy supply needed for effective immune responses, yet the specific mechanisms linking vitamin B5 metabolism to host defense remain unclear.

Vanin enzymes, particularly vanin-1, play a critical role in vitamin B5 metabolism by catalyzing the conversion of pantetheine into pantothenic acid and cysteamine. This pantothenic acid serves as a precursor for the biosynthesis and recycling of CoA. Beyond this metabolic role, the cysteamine produced through this reaction functions as a potent regulator of cellular redox status, enhancing tissue resilience to oxidative stress and contributing to the mitigation of inflammation (10–12). This dual metabolic function positions vanin enzymes at a unique intersection between vitamin B5 metabolism and cellular redox homeostasis.

Recent studies have highlighted the importance of redox regulation in viral infections, particularly in influenza infection (13). Viral infections trigger oxidative stress and significantly alter cellular redox homeostasis by disrupting antioxidant defense mechanisms (14). These changes in redox status play pivotal roles in viral pathogenesis, influencing both viral replication and host cell death pathways (13, 14). Given the dual role of vanin enzymes in vitamin B5 metabolism and redox regulation, understanding their function during viral infections could reveal new insights into how metabolic pathways influence host defense.

In this study, we explored how the vanin-1–mediated connection between vitamin B5 metabolism and redox regulation influences host defense during viral infection. Using a mouse model of influenza infection, we examined how vanin-1 deficiency affects survival, viral clearance, immune responses, and tissue homeostasis. Our findings reveal an unexpected role for vanin-1 in promoting host tolerance through modulation of cellular redox pathways, independent of its canonical function in vitamin B5 metabolism.

## Results

**Figure 1:**
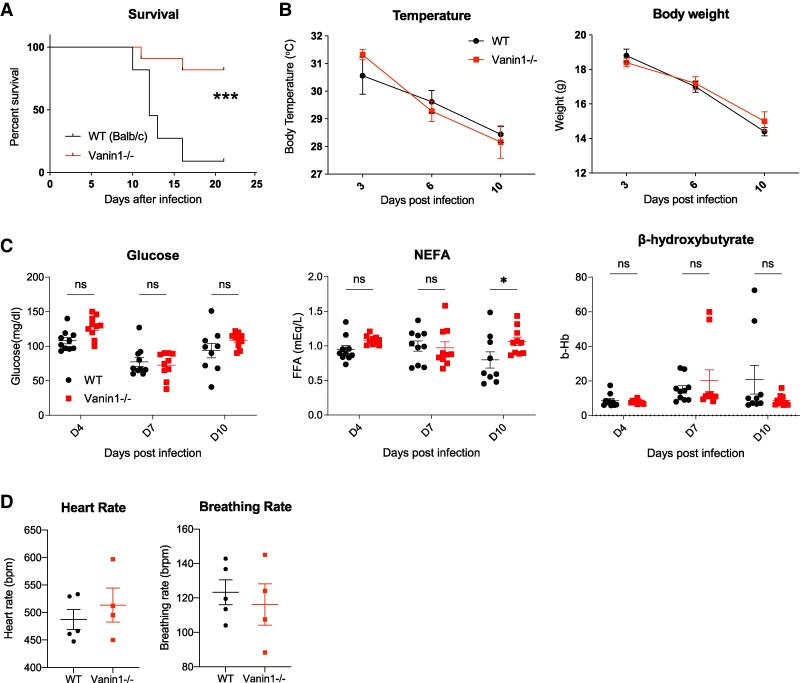
Vanin-1 deficiency led to the protection against influenza infection without affecting autonomic control and metabolism. A) Survival after influenza infection (*n* = 11/group, WSN 375 PFU, repeated three times). B) Core body temperature (left) and body weight change (right) during the course of influenza infection (*n* = 5/group). C) Glucose (left), nonesterified free fatty acid (middle), and β-hydroxybutyrate (right) during the course of influenza infection. D) Heart rate (left) and breathing rate (right) on day 7 postinfection of influenza virus. Data were presented as means ± SEM. Data were analyzed by Kaplan–Meier statistical analysis (A) and two-way ANOVA (C). ***P < 0.001, *P < 0.05, ns, not significant.

To investigate the role of vanin-1 in influenza infection, we compared responses to influenza virus (WSN) infection between wild-type (WT) and vanin-1 knockout (VKO) mice (15). Surprisingly, vanin-1–deficient mice showed significant protection against lethal influenza infection as demonstrated by enhanced survival (Fig. 1A). Despite this survival advantage, we observed no differences in morbidity markers, including core body temperature and body weight loss, between WT and VKO mice (Fig. 1B). Given that host tolerance to influenza infection can be affected by metabolic state and autonomic control (16), we examined these parameters in our model. Analysis of systemic metabolic markers revealed minimal changes in glucose, lipid, and β-hydroxybutyrate levels between WT and VKO mice during infection (Fig. 1C), with modest increase in nonesterified fatty acid (NEFA) levels in VKO mice at day 10 postinfection. Similarly, measurements of autonomic parameters showed no differences in heart rate and breathing rate between WT and VKO mice throughout the course of infection (Fig. 1D).

**Figure 2:**
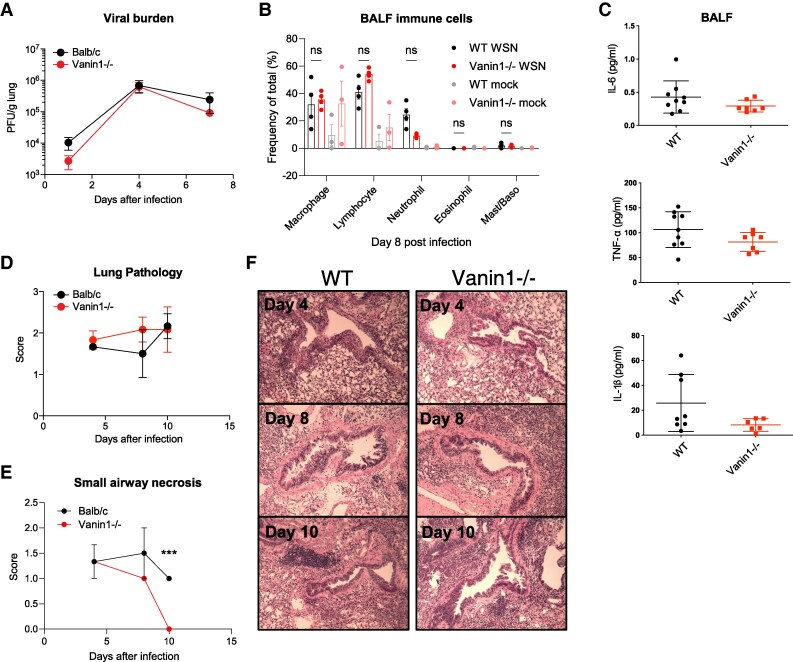
Protection against influenza infection with vanin-1 deficiency was not mediated by enhanced antiviral immunity nor reduced immunopathology. A) Plaque-forming assay from total lung lysates during the course of influenza infection (*n* = 3/group). B) Immune cell count in BAL fluid with cytospin and diffquick hematoxylin and eosin staining (*n* = 3–4/group). C) Cytokine measurement from BAL fluid at 5 days after infection. D) Lung pathology score after influenza infection (*n* = 6/group). E) Small airway necrosis score after influenza infection (*n* = 3/group). F) Histology of lungs after influenza infection. Data were presented as means ± SEM. Data were analyzed by two-way ANOVA (B and E). ns, not significant.

We next asked whether enhanced survival of VKO mice was mediated through changes in antiviral immunity or immunopathology. Analysis of viral burden showed no differences between WT and VKO mice during the course of infection (Fig. 2A). To assess the immune response in detail, we isolated immune cells from bronchoalveolar lavage (BAL) during infection and characterized immune cell populations. We found no major differences in immune cell counts between WT and VKO mice (Fig. 2B). Consistent with these cellular findings, levels of inflammatory cytokines, including interleukin (IL)-6, tumor necrosis alpha (TNF-α), and IL-1β in BAL fluid were comparable between WT and VKO mice during influenza infection (Fig. 2C). We next examined potential differences in tissue damage and inflammation. Comprehensive assessment of lung pathology throughout the course of infection also showed no significant differences between the groups, although VKO mice exhibited reduced small airway necrosis scores at day 10 postinfection (Fig. 2D–F). These findings demonstrate that the enhanced survival of VKO mice is not mediated by altered antiviral immunity or reduced immunopathology, pointing to the possibility of an alternative host defense mechanism.

**Figure 3:**
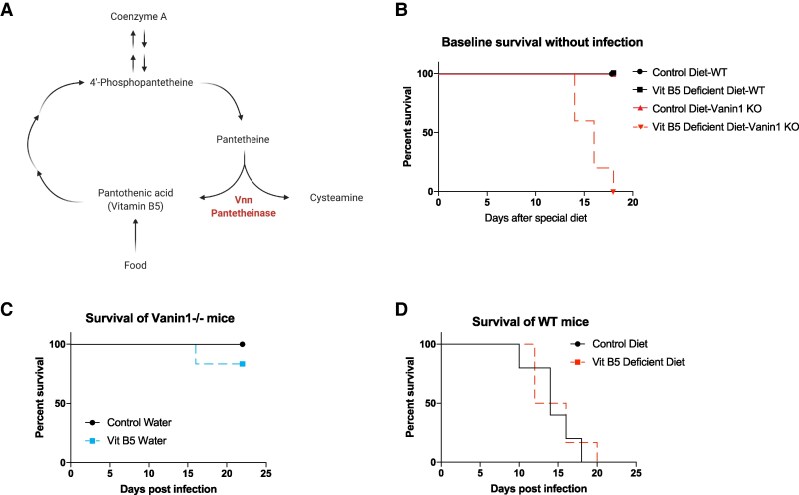
Vitamin B5 level did not affect the tolerance to influenza infection. A) Pantetheine metabolism controlled by vanin-1. B) Survival of mice treated with control diet or vitamin B5–deficient diet (Vit B5–deficient diet; *n* = 5/group, male mice). C) Survival of male vanin-1-/- mice with influenza infection (WSN, 450 PFU) with or without vitamin B5 supplementation (150 mM, 2 weeks before and during infection; *n* = 5–6/group, repeated three times). D) Survival of WT mice with influenza infection (WSN, 350 PFU) with control diet or vitamin B5–deficient diet (2 weeks before and during infection; *n* = 5–6/group, repeated four times). Data were presented as means ± SEM.

To understand the mechanism of protection in VKO mice, we first examined the role of vitamin B5 metabolism, the canonical pathway regulated by vanin-1. Vanin-1 functions as an ectoenzyme that controls vitamin B5 (pantothenic acid) levels by catalyzing the breakdown of pantetheine, which is subsequently recycled for coenzyme A synthesis (Fig. 3A). As expected from this metabolic role, VKO mice have been reported to have reduced levels of vitamin B5 (17). Consistent with this report, we found that VKO mice showed heightened sensitivity to vitamin B5 depletion, spontaneously succumbing to death under vitamin B5–deficient diet even in the absence of infection (Fig. 3B). Given the high intestinal expression of vanin-1, this severe phenotype may also reflect impaired processing of dietary CoA derivatives into pantothenate in VKO mice. Based on these observations, we speculated that decreased serum vitamin B5 levels might influence the survival of VKO mice during influenza infection. However, vitamin B5 supplementation failed to alter the survival advantage of VKO mice during influenza infection (Fig. 3C). Furthermore, vitamin B5 deficiency did not confer protection to WT mice against lethal influenza infection (Fig. 3D). These results indicate that the protective effect of vanin-1 deficiency during influenza infection may operate through a mechanism independent of vitamin B5 and its associated CoA metabolism.

**Figure 4:**
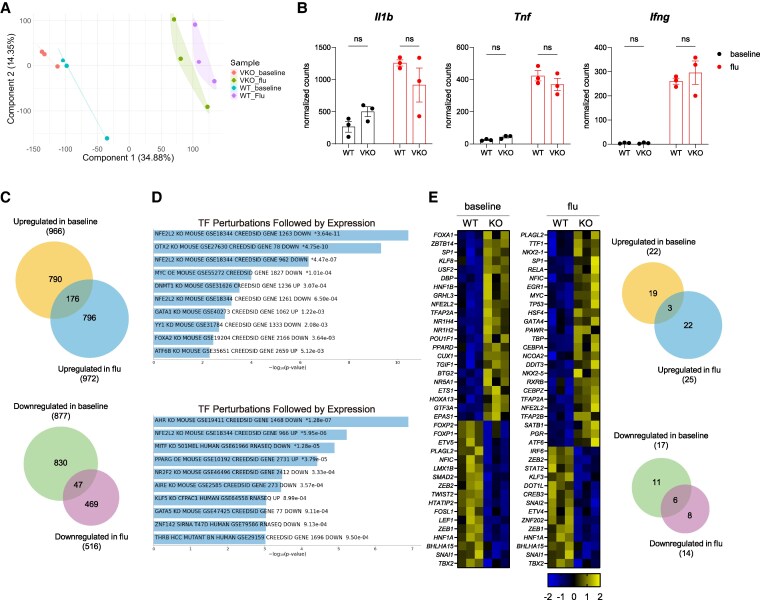
The transcriptional profiles of the vanin-1–deficient mice lungs are inversely correlated with Nfe2l2 activation. A) Principal component analysis (PCA) of transcriptomes of lungs 7 days after influenza infection (WSN 150 PFU) in WT and vanin-1–deficient mice (*n* = 3/group). B) Representative normalized counts of *Il1b*, *Tnf*, and *Ifng*. Data are represented as means ± SEM. C) Proportional Venn diagrams demonstrating overlap of genes up-regulated or down-regulated (*P* < 0.05) in vanin-1-/- lung in control (top) or infected (bottom) conditions. D) Enrichr “TF Perturbations Followed by Expression” analysis of up-regulated (top) and down-regulated (bottom) genes from overlaps of control and infected conditions in vanin-1-/- lungs. E) Heat map of *Z*-score for differentially activated transcription factors (*P* < 0.05) (left) and Venn diagram illustrating overlap of transcription factor activities in vanin-1-/- lungs across control or infected conditions (right). Data were analyzed by two-way ANOVA (B) and Student's t test (E). ns, not significant.

**Figure 5:**
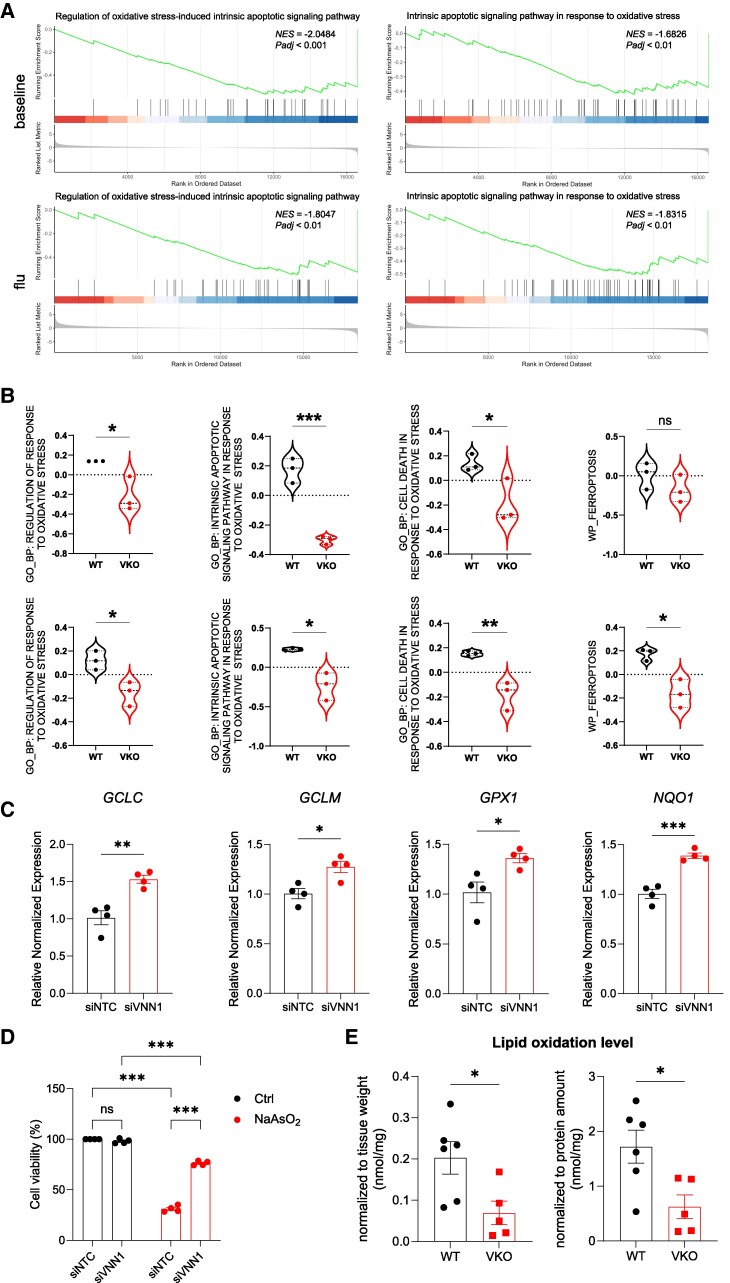
Vanin-1 deficiency protects against influenza infection–induced ferroptosis by inhibiting oxidative stress. A) GSEA plot of representative pathways (GO biological process) in vanin-1-/- lung compared with WT under control (top) and infected conditions (bottom; *n* = 3/group). B) Enrichment score from GSVA of representative pathways in control (top) and infected conditions (bottom; (*n* = 3/group). C) Relative RT-qPCR expression of *GCLC*, *GCLM*, *GPX1*, and *NQO1* transcript for A549 cells transfected with nontargeting siRNA (siNTC) or siVNN1 (*n* = 4/group). D) Relative cell viability from WST-8 assay of A549 cells treated with NaAsO_2_ under VNN1 silencing (*n* = 4/group). E) TBARS assay measuring MDA levels in lung tissues of WT and vanin-1-/- mice. MDA levels were normalized to tissue weight (left) or protein amount (right). Data were presented as means ± SEM. Data were analyzed by Student's t test (B, C, and E) and two-way ANOVA (D). ***P < 0.001, **P < 0.01, *P < 0.05, ns, not significant.

To understand the potential mechanism for this protection, we then conducted bulk RNA sequencing from the lung tissues of WT and VKO mice before and after influenza infection. Principal component analysis revealed that transcriptional profiles clustered primarily by infection status rather than vanin-1 deficiency (Fig. 4A). Consistent with the cytokine profile in BAL fluid, the infection-induced cytokine genes, such as *Il1b*, *Tnf*, and *Ifng*, were not different between WT and VKO (Fig. 4B). Further analysis of differential expression of genes (DEG) identified a substantial set of vanin-1–regulated genes: ∼1,800 genes in control conditions (966 up, 877 down) and 1,500 genes during infection (972 up, 516 down; Fig. 4C). This analysis also identified a subset of genes (176 up-regulated and 47 down-regulated) that remained consistently regulated by vanin-1 across both control and infected conditions. This shared gene signature represents the core molecular programs regulated by vanin-1. We then assessed potential upstream regulators of these core gene subset using the Enrichr database (18). Enrichr transcription factor perturbation analysis compares gene signatures with known transcriptional changes from various conditions of genetic perturbations. Genes up-regulated in VKO mice significantly matched those down-regulated in Nfe2l2 KO mice, while genes down-regulated in VKO mice corresponded to those up-regulated in Nfe2l2 KO mice (Fig. 4D). This inverse correlation suggests that vanin-1 deficiency activates Nfe2l2-dependent transcriptional programs. We further explored upstream transcription factors using decoupleR analysis with the complete RNA-seq dataset (19). The analysis revealed distinct transcription factor activities between WT and VKO mice, represented as a heat map showing differential regulatory patterns in both baseline and infected conditions (Fig. 4E). Among the differentially regulated transcription factors, three regulators (*Tfap2a*, *Nfe2l2*, and *Sp1*) consistently showed enhanced activity specifically in up-regulated gene sets from VKO mice across both baseline and infected conditions. Notably, Nfe2l2 was identified as a key regulator in both decoupleR and Enrichr analyses (Fig. 4E), which suggests its central role in vanin-1–dependent transcriptional regulation. Since Nrf2, a gene product of *Nfe2l2*, is the master regulator of antioxidant responses (20), we examined oxidative stress-related pathways. Gene set enrichment analysis (GSEA) of gene ontology biological process (GOBP) terms revealed that two related pathways on oxidative stress-induced apoptosis were significantly enriched in WT compared to VKO mice across both baseline and infected conditions (Fig. 5A). To validate our GSEA findings, we conducted gene set variation analysis (GSVA), which uses an unsupervised approach to measure pathway activity in each sample, providing a clearer view of how pathways vary across the dataset (21). In agreement with GSEA results, response to oxidative stress and its related cell death pathways were significantly reduced in VKO mice across both baseline and infected conditions (Fig. 5B). Additionally, the ferroptosis pathway, a form of oxidative stress-induced cell death, was specifically down-regulated in VKO mice during influenza infection (Fig. 5B). These findings align with previous reports of enhanced antioxidant pathways in vanin-1–deficient mice (10), where increased glutathione (GSH) synthesis was observed in the liver. To examine whether similar mechanisms operate in lungs, we knocked down vanin-1 in lung epithelial cells and found significant up-regulation of genes involved in GSH metabolism, including *GCLC* and *GCLM* (biosynthesis enzymes), as well as *GPX1* and *NQO1* (GSH-dependent detoxification enzymes; Fig. 5C). Given the emerging role of ferroptosis, a form of oxidative stress-induced cell death in influenza pathogenesis (22, 23), we investigated whether vanin-1 deficiency protected against ferroptosis. Indeed, vanin-1 knockdown significantly reduced sodium arsenite-induced ferroptosis (Fig. 5D). Supporting these findings, VKO mice showed decreased lipid peroxidation in their lungs (Fig. 5E).

**Figure 6:**
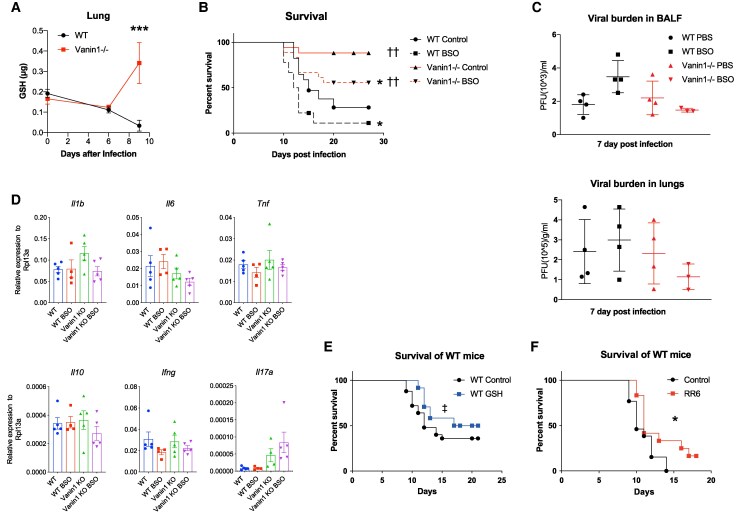
Inhibition of antioxidant pathway was sufficient to reduce tolerance associated with vanin-1 deficiency to influenza infection. A) GSH level in lungs in the course of influenza infection (*n* = 6/group). B) Effect of GSH synthesis inhibition with BSO on survival against influenza infection (WSN 350 PFU, *n* = 17–18/group, repeated three times). C) Viral burden in BAL fluid (BALF, top) and total lungs (down) 7 days after influenza infection with or without BSO treatment. D) Gene expression analysis with quantitative PCR in total lungs 7 days after influenza infection with or without BSO treatment. E) Survival of WT (Balb/c mice) after influenza infection (WSN 350 PFU) with or without GSH supplementation in food (*n* = 24/group). F) Survival of WT mice after influenza infection (WSN 375 PFU) with or without vanin-1 inhibitor (RR6) treatment (*n* = 12/group). Data were presented as means ± SEM. Data were analyzed by two-way ANOVA (A) and Kaplan–Meier statistical analysis (B, E, and F). ***P < 0.001, *P < 0.05 vs. control, ††*P* < 0.01 vs. WT, ‡*P* = 0.064 vs. WT control.

Based on these findings, we next investigated how enhanced antioxidant capacity influences influenza infection outcomes in vivo. During lethal influenza infection, VKO mice maintained significantly higher GSH levels in their lungs, particularly at the late stage of infection (Fig. 6A). Consistent with this antioxidant protection, VKO mice exhibited significantly reduced small airway necrosis scores at day 10 postinfection (Fig. 2E). To determine whether enhanced GSH synthesis mediates this protection, we treated mice with buthionine sulfoximine (BSO), a GSH synthesis inhibitor. BSO treatment significantly reduced survival in both VKO and WT mice during lethal influenza infection (Fig. 6B), demonstrating that enhanced antioxidant capacity was indeed the key protective mechanism in VKO mice. BSO treatment did not affect viral burden (Fig. 6C) and inflammatory cytokine expression in lungs (Fig. 6D), indicating that the antioxidant pathway specifically influences survival independent of host resistance. Supporting this protective role, dietary GSH supplementation was sufficient to protect WT mice from lethal influenza infection (Fig. 6E). To explore the therapeutic potential of our findings, we next tested whether pharmacological inhibition of vanin-1 could protect against lethal influenza infection. Indeed, treatment with a vanin-1 inhibitor provided similar protection as genetic deletion (Fig. 6F), validating vanin-1 as a potential therapeutic target in severe influenza infection. Together, these findings demonstrate that vanin-1 deficiency enhances survival during severe influenza infection through GSH-dependent antioxidant defense.

## Discussion

In this study, we demonstrate that vanin-1 deficiency enhances host tolerance to lethal influenza infection through modulation of cellular redox status. Despite vanin-1's established role in vitamin B5 and CoA metabolism, neither vitamin B5 supplementation nor deficiency affected survival during infection. Instead, our transcriptional analysis revealed activation of Nrf2-dependent antioxidant pathways in VKO mice, particularly enhanced GSH synthesis. The functional significance of this enhanced antioxidant capacity was evident through reduced lipid peroxidation and protection against ferroptosis during infection. We confirmed this mechanism through both loss- and gain-of-function approaches: pharmacological inhibition of GSH synthesis with BSO abolished the survival advantage in VKO mice, while GSH supplementation protected WT mice from lethal infection. Importantly, this protection occurred independently of viral clearance, inflammatory responses, or metabolic processes, indicating that vanin-1 regulates host tolerance specifically through redox-dependent tolerance mechanisms rather than canonical resistance pathways.

Host tolerance mechanisms can be regulated by diverse metabolic pathways, as demonstrated by the key roles of glucose availability, ketone body production, and triglyceride metabolism during infection (2, 16). Vanin-1 produces two major products—pantothenic acid for CoA synthesis and cysteamine, a small thiol compound known to influence cellular redox status. The unexpected role of vanin-1 in regulating host tolerance through redox status, rather than through its canonical vitamin B5 metabolism, reveals a novel function for this enzyme. Although a previous study showed that cysteamine reduced replication of the influenza virus in vitro, our results in mouse models suggest that vanin-1 regulates host tolerance through redox regulation rather than altered viral clearance in vivo (24). The relative importance of these pathways may depend on disease model, tissue context, and timing, as highlighted in previous studies (25). While vanin-1's role in vitamin B5 and CoA metabolism is essential for survival under nutrient-deficient conditions, this metabolic function appears dispensable during infection. Of note, the severe intolerance of VKO mice to a vitamin B5–deficient diet may also reflect impaired processing of food-derived CoA intermediates in the intestine, where vanin-1 is highly expressed, in addition to loss of endogenous CoA recycling. Our findings reveal that vanin-1 regulates host tolerance primarily through redox regulation rather than metabolic control during severe infection. Previous studies suggested that cystamine, converted from cysteamine, reduces cellular GSH by inhibiting γ-glutamylcysteine synthetase activity (10). While this mechanism remains valid, our findings demonstrate that vanin-1 deficiency also enhances transcription of GSH synthetic enzymes, revealing an additional layer of redox regulation.

Viral infections, particularly influenza, significantly alter cellular redox homeostasis by triggering oxidative stress and disrupting antioxidant defense mechanisms. This is especially critical as the influenza virus actively suppresses antioxidant pathways, including G6PD, SIRT2, and NRF2, promoting viral replication through increased oxidative stress (26). This virus-induced oxidative stress leads to respiratory epithelial cell death, while antioxidant treatment can prevent both cellular death in vitro and lung damage in vivo (27, 28). Our findings demonstrate that vanin-1 deficiency maintains redox homeostasis through enhanced GSH synthesis and Nrf2-dependent antioxidant programs, ultimately protecting against oxidative stress-induced cell death, as evidenced by reduced small airway necrosis. Importantly, we identified protection against ferroptosis as a novel mechanism of enhanced host tolerance in vanin-1–deficient mice. This finding is further supported by recent studies highlighting the pathogenic role of ferroptosis in severe influenza infection (22, 23).

Our findings identify vanin-1 as a potential therapeutic target for enhancing host tolerance during severe influenza infection. We demonstrate that pharmacological inhibition of vanin-1, similar to genetic deletion, protected mice against lethal infection. Given that this protection operates through enhanced antioxidant defense rather than viral control, targeting vanin-1 may provide a complementary strategy to current antiviral approaches, particularly in severe cases where oxidative damage drives mortality. This mechanism of enhanced host tolerance through redox regulation may have broader implications for other viral infections where oxidative stress contributes to disease severity. However, since our experiments were conducted in systemic Vnn1–deficient mice on a BALB/c background, and Vnn1-dependent phenotypes can vary depending on genetic background and disease context (25), further studies will be needed to confirm these findings in other models.

## Materials and methods

### Mice

All animal experiments were conducted in compliance with institutional guidelines following protocol review and approval by Yale University's Institutional Animal Care and Use Committee. Unless specified otherwise, male and female Balb/cJ mice (The Jackson Laboratory Stock No. 000651) or vanin-1–deficient mice, aged 7 to 10 weeks, were utilized for the experiments. For mouse infections, the influenza virus strain A/WSN/33, originally obtained from Dr Akiko Iwasaki's laboratory, was propagated in Madin–Darby Canine Kidney (MDCK) cells and its concentration determined using a plaque assay as described below. During influenza infections, mice were anesthetized with a ketamine/xylazine mixture, and the specified number of plaque-forming units (PFUs) of the virus in 30 μL PBS was administered intranasally dropwise, as previously described (16). Respiration and heart rates were monitored using pulse oximetry with the MouseOx Plus (Starr Life Sciences Corp.), while core body temperature was measured using a rectal probe thermometer (Physitemp TH-5 Thermalert).

For dietary GSH supplementation, mice were given GSH (final concentration of 22 mg/mL in drinking water) 4 h before infection and continued on treatment until the end of the experiment, with drinking water replaced every day. For pharmacological inhibition of vanin-1, mice were given RR6 (final concentration of 1.2 mg/mL in drinking water) 4 h before infection and continued on treatment until the end of the experiment, with drinking water replaced every 4 days.

### Quantification of viral loads

To assess the titers of influenza virus strain A/WSN/33 in the lungs, lung tissue was collected at specified time points postinfection, weighed, and disrupted using bead homogenization. For determining viral titers in BAL fluid, mice were euthanized, and the trachea was exposed. BAL was performed by cannulating the trachea and lavaging the lungs with 1 mL of PBS. Virus titers were measured by infecting MDCK cells with serially diluted lung homogenate and BAL fluid, followed by the application of an agar overlay for 48 h. Subsequently, cell monolayers were stained with crystal violet, and the number of plaques was counted.

### Histological analysis

All procedures were performed with minor modifications from our previous report (16). All mice were euthanized via carbon dioxide asphyxiation, followed by perfusion with either PBS or a fixative. Tissues were immersion-fixed using 10% neutral buffered formalin. Standard procedures were applied to trim, process, embed, section, and stain tissues with hematoxylin and eosin. Pathological scores were assessed while blinded to both experimental and genetic conditions. Digital light microscopy images were captured using a Zeiss Axio Imager A1 microscope equipped with an AxioCam MRc5 camera and AxioVision 4.8.3.0 imaging software (Carl Zeiss MicroImaging, Inc.).

### RNA sequencing and analysis

For tissue RNA extraction, lung tissues were harvested into easy-BLUE Total RNA Extraction Kit (iNtRON Biotechnology, Korea) and disrupted by bead homogenization in screw tubes (ALLSHENG, China) with zirconia ceramic beads (LABOTEC, Korea) using a Bioprep-6 homogenizer (ALLSHENG). RNA was extracted using the RNeasy Kit, according to the manufacturer's protocol (QIAGEN). RNA-seq libraries were constructed following the Illumina TruSeq stranded mRNA Reference Guide #1000000040498 v00. Paired-end sequencing was performed with NovaSeqX with 101 bp reads from each end. Reads were aligned to the mouse genome mm39 by STAR v2.7.10a and quantified using featureCounts v2.0.3. Raw counts were normalized, and differentially expressed genes between WT and VKO mice were identified using DESeq2 v1.42.1. Gene ontology enrichment analysis was performed using clusterProfiler v4.2.2 with enriched pathways from the Molecular Signature Database (MSigDB) (29) on gene lists ranked by DESeq2 results, and the results were filtered for FDR<0.05 with a minimum gene set size of 10. Gene set variation analysis was further used to estimate scores of given gene sets in each sample with GSVA v1.42.0 (21) using normalized counts generated from DESeq2. The transcription factor (TF) perturbation assay was conducted on common DEGs from baseline and flu using the Enrichr's TF Perturbations Followed by Expression database (18), which contains gene expression signatures from experimental TF perturbations. TF activity inference was generated by decoupleR v2.8.0 (19). DESeq2 normalized counts were analyzed with the weighted mean (WMEAN) and univariate linear regression approaches to compute activity scores. To filter out low expression genes in TF activity inference, we only used genes with count over 50 for the analysis.

### Cell culture and knockdown study

Lung epithelial cell A549 was obtained from the Korean Cell Line Bank. Cells were cultured with RPMI 1640 (Welgene, Korea) supplemented with 10% fetal bovine serum (FBS) (Gibco) and 1% penicillin–streptomycin (Gibco). The cells were maintained at 37 °C with 5% CO_2_ under humidified conditions. For vanin-1 knockdown study, A549 cells were seeded on 24-well plates (SPL, Korea) and allowed to adhere overnight. On the next day, cells were transfected with either siVNN1 or nontargeting siRNA (Bioneer, Korea) using RNAiMAX (Invitrogen, USA) in serum-free Opti-MEM (Gibco), according to the manufacturer's protocols. The sequence of siRNA for VNN1 is listed in Table S1.

### RNA extraction and quantification

Total RNA from cultured cells was extracted using phenol-chloroform extraction with easy-BLUE Total RNA Extraction Kit, according to the manufacturer's protocol. cDNA synthesis was performed using MMLV reverse transcriptase (CellSafe, Korea) with oligo(dT) primers. qRT-PCRs were performed on a CFX Opus 96 Real-Time System (Bio-Rad) using HiSense QGreenBlue qPCR Master Mix (CellSafe, Korea) containing SYBR Green type dye, and transcript levels were normalized to GAPDH. Primers used for qRT-PCR are listed in Table S1.

### BAL analysis and cytokine and metabolites measurement

BAL was conducted in euthanized animals by cannulating the trachea and flushing the lungs with 1 mL of PBS. For BAL cell counts, collected cells were centrifuged onto glass slides using a Cytospin 4 (Thermo Scientific), and stained samples were examined under a Zeiss Axioplan2 microscope. Cytokine levels of IL-6, IL-1β, and TNF-α in BAL fluid were quantified via sandwich enzyme-linked immunosorbent assay, employing capture antibodies (eBioscience and BD), biotin-conjugated detection antibodies (eBioscience and BD), HRP-conjugated streptavidin (BD), and TMB substrate reagent (BD). Plasma β-hydroxybutyrate levels were determined using a commercial kit following the manufacturer's instructions (Cayman Chemical). Plasma NEFA concentrations were assessed with a kit, according to the manufacturer's protocols (Wako Diagnostics). Capillary blood glucose was measured using a BD OneTouch Ultra 2 blood glucose monitor, following the manufacturer's guidelines.

### Cell viability assay under oxidative stress

Cells were seeded on 96-well plates then treated with 100 μM sodium arsenite (Sigma-Aldrich, USA) (30) to induce ferroptotic cell death. Cell viability was assessed 24 h after the treatment using a WST-8 assay kit (Biomax, Korea). Cell culture medium was replaced with fresh medium containing WST-8 reagent and incubated for 1 h in a 5% CO_2_ incubator at 37 °C. The cell viability was expressed as absorbance values at 450 nm measured with a microplate reader (Tecan, Switzerland). Cell viability was calculated as a percentage relative to control cells, which were defined as 100%.

### Lipid peroxidation assay

For colorimetric assay of lipid peroxidation, malondialdehyde (MDA) levels were measured from lung tissues of WT and VKO mice using OxiTec Thiobarbituric Acid Reactive Substances (TBARS) Assay kit (Biomax, Korea), according to the manufacturer's protocols. Frozen tissues were weighed and mechanically lysed in radio-immunoprecipitation assay buffer (Biosesang, Korea) supplemented with Xpert Duo Inhibitor Cocktail Solution (GenDEPOT, USA) using a bead beater. Lysates were centrifuged (13,000 rpm, 10 min, 4 °C), and supernatants were used for the assay. The absorbance at 530 nm was measured to determine the MDA concentration, which was normalized by either tissue weight or total protein amount. Protein concentrations of lung lysates were determined by a BCA assay kit (Biomax, Korea), according to the manufacturer's protocols.

### Statistical analysis

Statistical information, including *n*, mean, and statistical significance values, are indicated in the text or the figure legends. Data were statistically analyzed using Student's t test, or an ANOVA test with multiple comparisons where appropriate using Prism 10 (GraphPad Software, Inc., USA). Kaplan–Meier survival curves were compared using log-rank Mantel–Cox test. Comparisons with a *P*-value of <0.05 were considered statistically significant.

## Supplementary Material

pgag222_Supplementary_Data

## Data Availability

The sequencing data have been deposited in the NCBI Gene Expression Omnibus (GEO) under accession number GSE298514.
